# Supplementary material for: The structure and evolutionary diversity of the fungal E3-binding protein
Source: Commun Biol. 2023 May 3;6:480. doi: 10.1038/s42003-023-04854-7 (PMC10156792; doi:10.1038/s42003-023-04854-7)
Supplement: Supplementary file 2 — Supplementary Information [file 42003_2023_4854_MOESM2_ESM.pdf]

Supplementary information
to
"Structure and evolutionary diversity of the fungal E3-binding protein"

Bjoern. O. Forsberg[1,2]

[1]Department of Physiology and Pharmacology, Karolinska Institutet, Biomedicum, Solnavägen 9, 171 77 Stockholm, Sweden.

[2]Division of Structural Biology, Wellcome Centre for Human Genetics, University of Oxford, OX3 7BN Oxford, UK (present address).

Correspondence: bjorn.forsberg@ki.se

Supplementary Note 1

Symmetry expansion

Symmetry expansion utilized a custom icosahedral expansion matrix compatible with sym=T to allow easy C3-refinement following re-boxing. `relion_particle_symmetry_expand` was provided a symmetry file with the relion I2 rotation matrix rotated to match the relion T-symmetry though its `--sym` flag:

```
# IcosaCompatWithDefaultTetra.sym
rot_axis  2  0  0.816496  0.57735
rot_axis  3  0  0  1
rot_axis  5  -0.3717480344  0.9091823044  0.1875924741
```

Such a file can in theory be provided to `relion_refine`, but this an unsupported feature that will not work without further modifications to the code, since the removal of the redundant portion of the orientational sampling space is only defined for the predefined symmetries in RELION. A work-around that will by-pass this issue without fixing it, is to assign C1-symmetry for the sampling but the specified symmetry during reconstruction. This is not recommended. Hence, the present work only utilized predefined symmetries during refinement, and instead defined an expansion symmetry that was compatible with the tetrahedral symmetry. NOTE: The "revert and re-point polish bin1"-step involves 1) reverting the classified subset of the subtracted and re-centered particles to point to the polished bin2 full-core particles, 2) re-running the previous polishing step and output at bin1, 3) edit the reverted file and relevant fields of the optics group to reference the new polish bin1 job, 4) run a refinement using the edited file starting local searches, and 5) subtracting the polished bin1 particles using the new refinement. In essence, this permits classification at bin2 and subsequent up-scaling to bin1. This was necessary due to the high particle number after expansion in combination with particle subtraction, which generates a new images stack with one particle per alignment, as opposed to one particle per original un-expanded particle.

Supplementary Tables

Dataset	tE2+PX261	Native (EMPIAR-10489)
Carbon support	No	Yes
Microscope	FEI Krios	FEI Talos
Detector	Gatan K2 GIF	Falcon 2
Voltage	300 kV	200 kV
Magnification	165k (0.86Å/pix)	112k (1.25Å/pix)
Mode	Counting	Counting
Gain normalized	Yes	Yes
Micrographs	4063	4867
Total dose	31.4 e/Å^2, 4s	35.0 e/Å^2
Fractions	32	19

Supplementary Table 1: **Datasets collected.** No new data was collected, and these are collection parameters as described in Forsberg et al[1]. Native PDC data are available through EMPIAR-10489[2]

Accession	Protein[1]	Annot[2]	Species	Subkingdom	Division/phylum	Subdivision/subphylum	Class
RMX77418	E3BP	hypo	Hortaea werneckii	Dikarya	Ascomycota	Pezizomycotina	Dothideomycetes
XP017996629	PX	hypo	Phialophora attinorum	Dikarya	Ascomycota	Pezizomycotina	Eurotiomycetes
XP018001728	PX	PX	Phialophora attinorum	Dikarya	Ascomycota	Pezizomycotina	Eurotiomycetes
XP044666154	PX	unchar	Bacidia gigantensis	Dikarya	Ascomycota	Pezizomycotina	Lecanoromycetes
PBP15521	E2?	PX	Diplocarpon rosae	Dikarya	Ascomycota	Pezizomycotina	Leotiomycetes
KAF3097484	PX	pyridox	Orbilia oligospora	Dikarya	Ascomycota	Pezizomycotina	Orbiliomycetes
RPA78695	PX	hypo	Ascobolus immersus	Dikarya	Ascomycota	Pezizomycotina	Pezizomycetes
RPB21680	PX	PX	Terfezia boudieri	Dikarya	Ascomycota	Pezizomycotina	Pezizomycetes
XP956161	PX	PX	Neurospora crassa	Dikarya	Ascomycota	Pezizomycotina	Sordariomycetes
Q5AKV6	PX	hypo	Candida albicans	Dikarya	Ascomycota	Saccharomycotina	Saccharomycetes
P16451	PX	PX	Saccharomyces cerevisiae	Dikarya	Ascomycota	Saccharomycotina	Saccharomycetes
XP019021831	PX	unchar	Saitoella Complicata	Dikarya	Ascomycota	Taphrinomycotina	incertae sedis
XP018226045	PX	hypo	Pneumocystis carinii	Dikarya	Ascomycota	Taphrinomycotina	Pneumocystidomycetes
O94709	PX	PX	Schizosaccharomyces pombe	Dikarya	Ascomycota	Taphrinomycotina	Schizosaccharomycetes
XP002172135	PX	PX	Schizosaccharomyces japonicus	Dikarya	Ascomycota	Taphrinomycotina	Schizosaccharomycetes
XP041229627	PX	PX	Suillus Fuscotomentosus	Dikarya	Basisomycota	Agaricomycotina	Agaricomycetes
KAG9290904	PX	hypo	Geosiphon pyriformis	Zygomyceta	Glomeromycota	Glomeromycotina	Glomerales
CAG8600220	PX	-	Paraglomus brasilianum	Zygomyceta	Glomeromycota	Glomeromycotina	Paraglomerales
KAG0028626	PX	hypo	Podila clonocystis	Zygomyceta	Mucoromycota	Mortierellomycotina	Mortierellomycetes
CEJ03135	PX?	acyltr.	Rhizopus microsporus	Zygomyceta	Mucoromycota	Mucoromycotina	Mucorales
KAG2190366	PX	hypo	Mucor plumbeus	Zygomyceta	Mucoromycota	Mucoromycotina	Mucorales
KAG2189189	PX	hypo	Umbelopsis vinacea	Zygomyceta	Mucoromycota	Mucoromycotina	Umbelopsidomycetes
OLY84536	PX	PX	Smittum mucronatum	Zygomyceta	Zoopagomycota	Kickxellomycotina	Harpellales
PIA15600	PX	hypo	Coemansia reveresa	Zygomyceta	Zoopagomycota	Kickxellomycotina	Kickxellomycetes
KXN72557	PX	hypo	Conidiobolus coronatus	Zygomyceta	Zoopagomycota	Entomophthoromycotina	Entomophthoromycetes

Supplementary Table 2: **Modeled sequences** List of E3BP proteins modeled computationally using colabFold, as depicted in Supplementary Fig. 9, available through Zenodo 7801353[3]. [1]Gene designation in uniprot. [2]Gene product annotation at the time of publication.

	CBD trimer with core	CBD dimer with core	CBD monomer in solution
box dims [nm]	18.3, 18.5, 13.5	18.3, 18.5, 13.5	10.9, 10.9, 10.9
box angles [deg]	90.0 90.0 61.1	90.0 90.0 61.1	90.0, 90.0, 90.0,
atoms	394 272	392 351	128 428
Protein	55 593	53 068	2 525
TIP3P	337 884	338 487	125 658
Na (mM)	363 (179 mM)	367 (181 mM)	119 (158 mM)
Cl (mM)	432 (212 mM)	429 (211 mM)	126 (167 mM)
replicas	8	8	23
min t_{sim} [ns]	100	100	100
dt [fs]	2	2	2
Cutoff	Verlet	Verlet	Verlet
coulomb [nm]	1.0	1.0	1.0
vdw [nm]	1.0	1.0	1.0
barostat	P.R. semi	P.R. semi	P.R. semi

Supplementary Table 3: **List of simulations** Generation of salt ions used GROMACS genion with -conc 0.15 -neutral. Provided molar concentration of ions was calculated based on the provided atom numbers, and not volumetrically based on box dimensions. SImulation input files and simulation trajectories are available through Zenodo 7801353[3].

Supplementary Figures

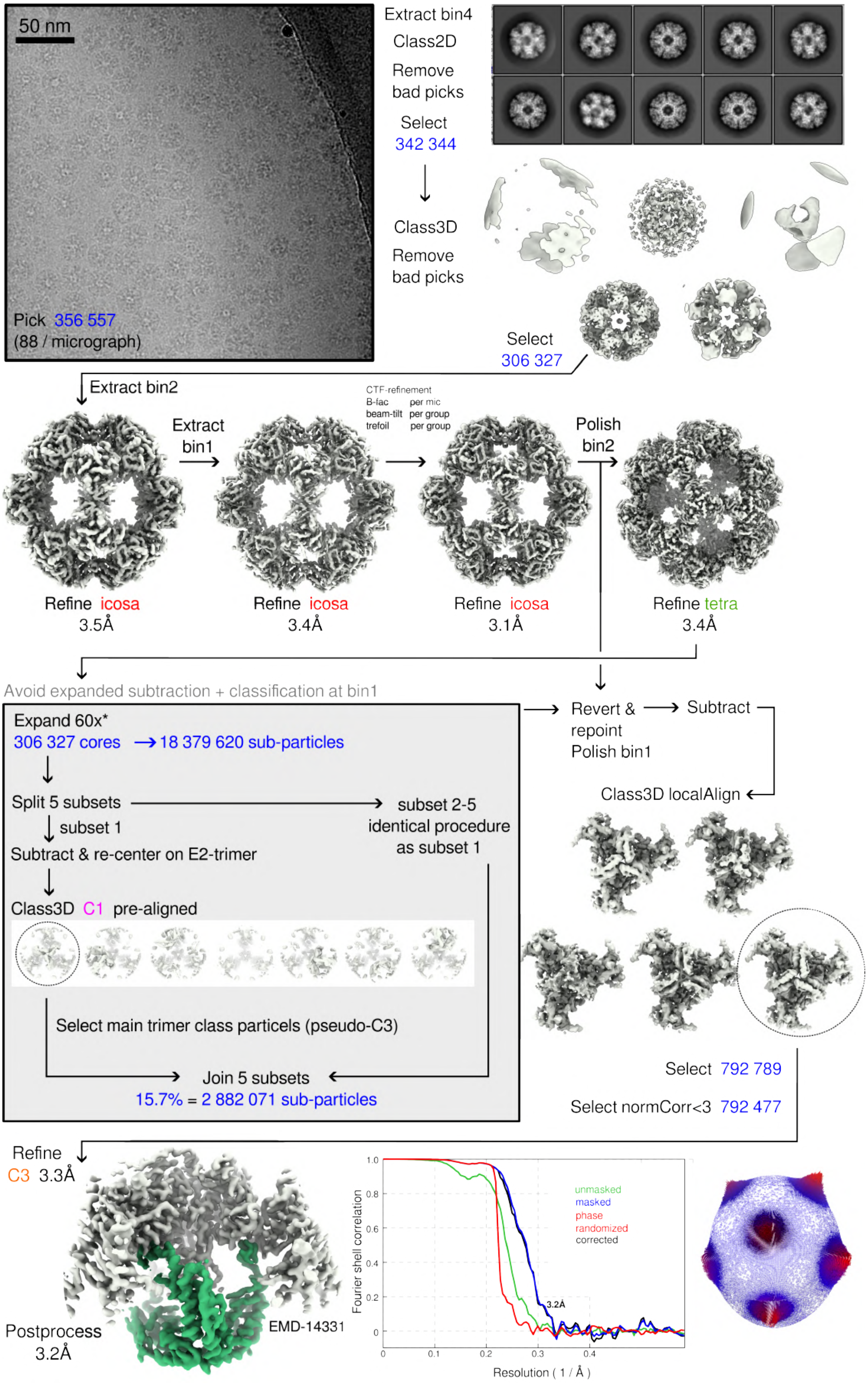

Supplementary figure 1: **Cryo-EM data processing details of recombinant E2-E3BP complex.** The processing pipeline is represented from top to bottom. Particle numbers are highlighted in blue, and percentages are relative to the number following 60x symmetry-expansion. Annotations in gray clarify the motivation of each step. Gold-standard FSC is shown, as established through RELIONs post-processing procedure. The orientational distribution following the final 3D-refinement under C3-symmetry shows 4 clusters in the asymmetric unit of the orientational plot, indicating that 5-fold faces are most prevalent in the original data, as is also perceived from micrograph images and 2D-classes.

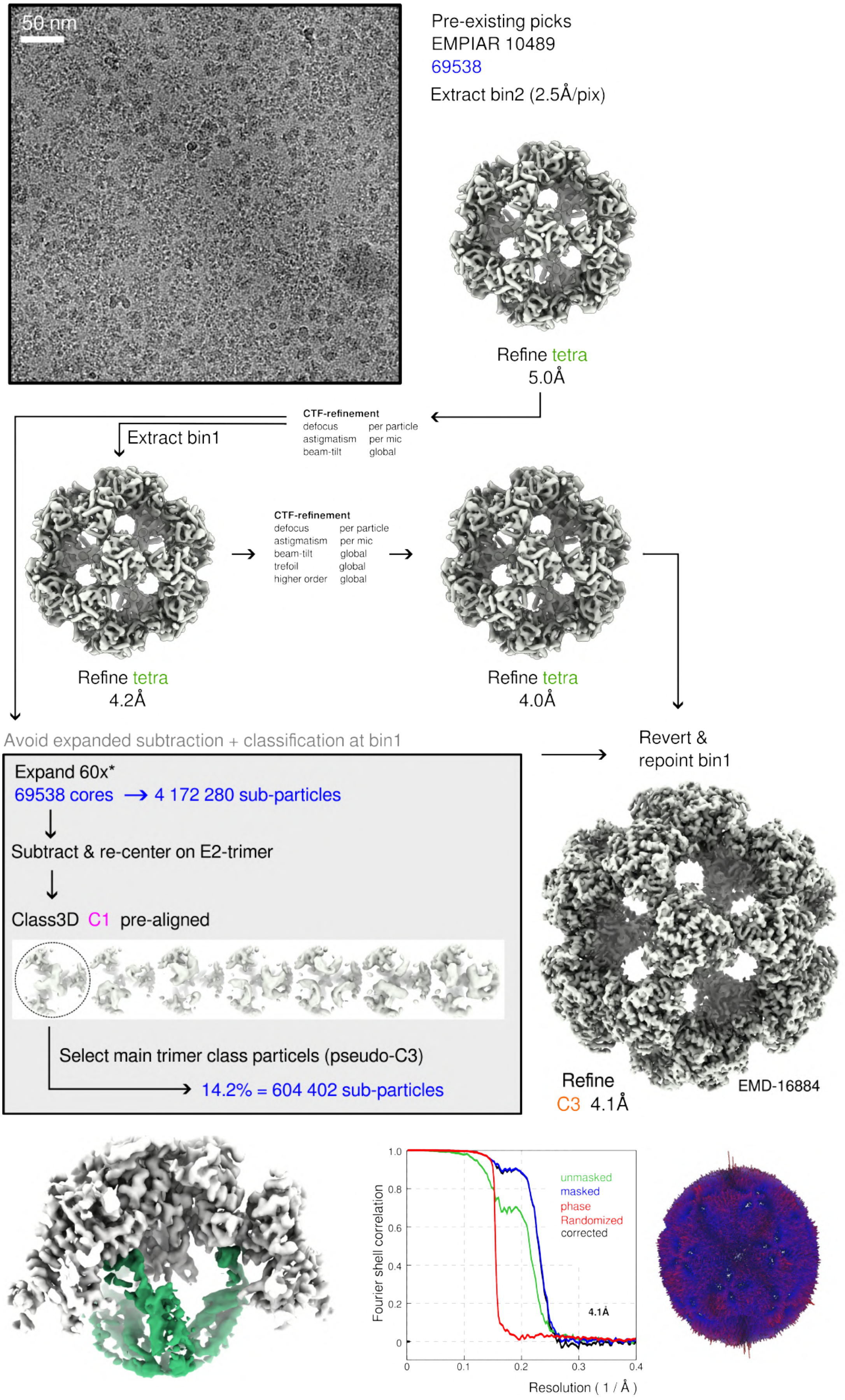

Supplementary figure 2: **Cryo-EM data processing details of native E2-E3BP sub-complex.** The processing pipeline is represented from top to bottom. Particle numbers are highlighted in blue, and percentages are relative to the number following 60x symmetry-expansion. Annotations in gray clarify the motivation of each step. Visual representations of 3D-classifications are by central slice in orthogonal views. The final reconstruction is shown as a surface representation, showing E3BP in green and E2 core in white. Gold-standard FSC is shown, as established through RELIONs post-processing procedure. The orientational distribution following the final 3D-refinement under C3-symmetry shows a uniform distribution, contrasting that of the recombinant sub-complex, and attributed to the flexible proteins surrounding the core which reduce influence of any specific interaction that would lead to non-uniform such distribution.

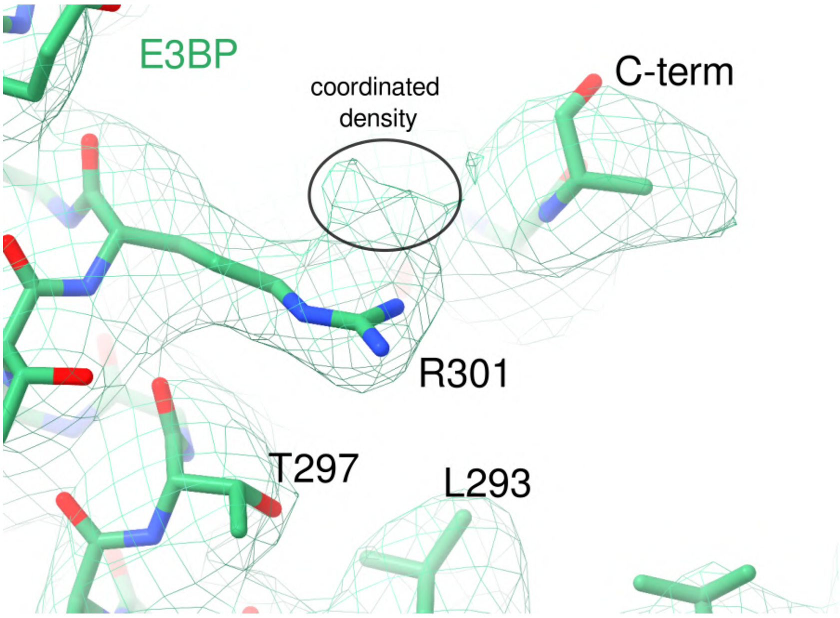

Supplementary figure 3: **C-terminal coordination by R301.** R301 is one of few near-universally conserved residues in fungal E3BP, and seems fully coordinated in the present reconstruction. Given the surrounding hydrophobicity that stabilizes both the E3BP monomer fold and the homomeric trimer point of contact, the only option for such coordination appears to be the C-terminal carboxyl group of A426. R301 does show additional density to imply such a coordination, however the resolution does not permit unambiguous placement of the 2 C-terminal residues. The penultimate residue of *N.crassa* E3BP is unusual by R425 (not shown), which is almost exclusively small and hydrophobic. This may incur increased flexibility to cause the reduced resolution in the present reconstruction.

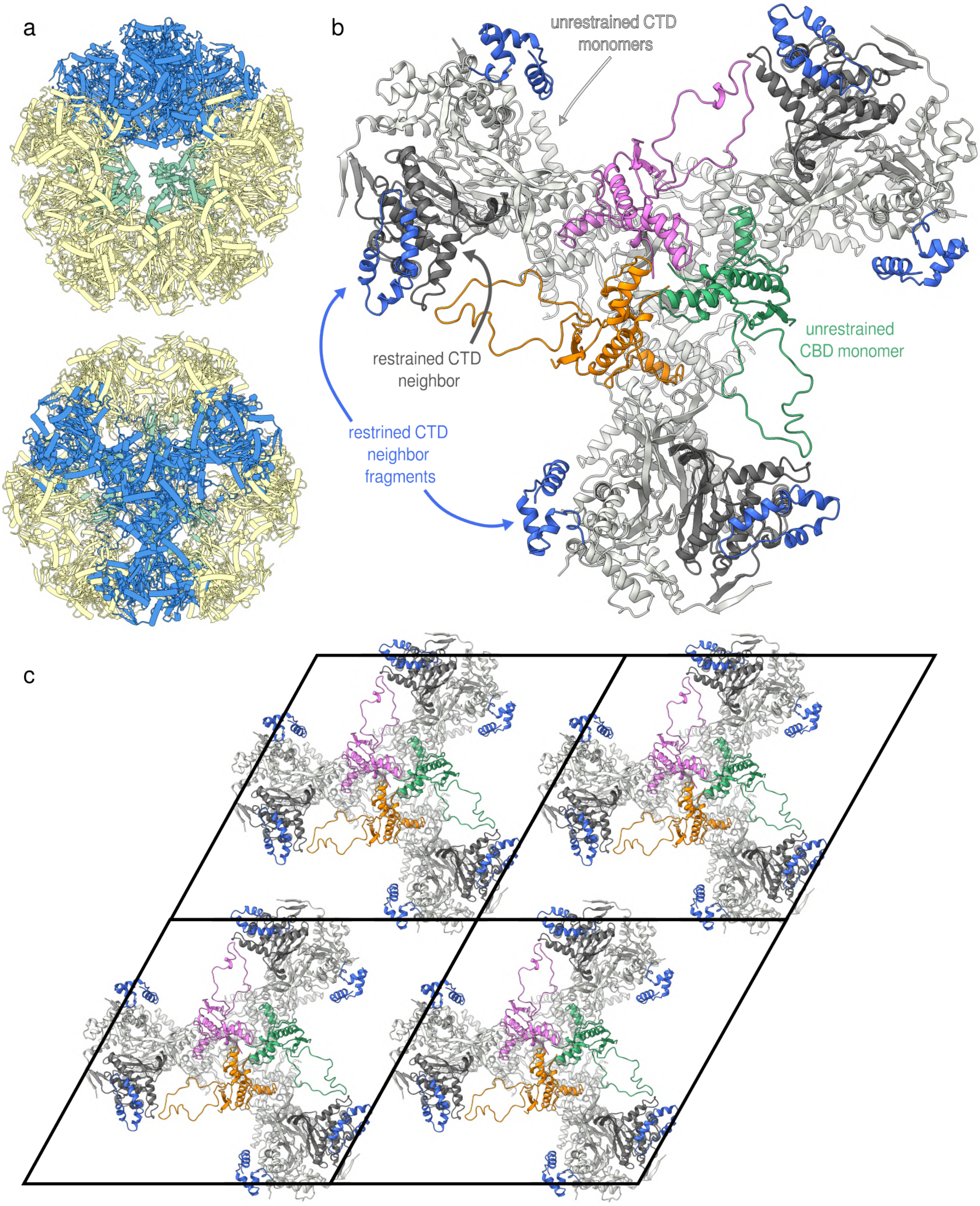

Supplementary figure 4: **Simulation setup.** **a**, The assembled PDC core of PDB entry 6ZLO was used as a template, and used to fit the E2 CTD and associated E3BP CBD of the present work (PDB 7R5M) into its asymmetric unit. The M3 loop was built in an unspecific conformation, without consideration for any density support. Following symmetry expansion to complete the PDC core, the region indicated in blue was then isolated and used for subsequent simulations. **b**, The neighbor fragments indicated in blue, and the neighbor CBD chains indicated in black were restrained at C-alpha atoms to emulate the influence of a semi-rigid core. All CTD chains indicated in white, and all CBD chains (pink, orange and green) were unrestrained in all simulations. **c**, The periodic box used to simulate molecular dynamics under semi-isotropic pressure coupling and absolute position restrains as described.

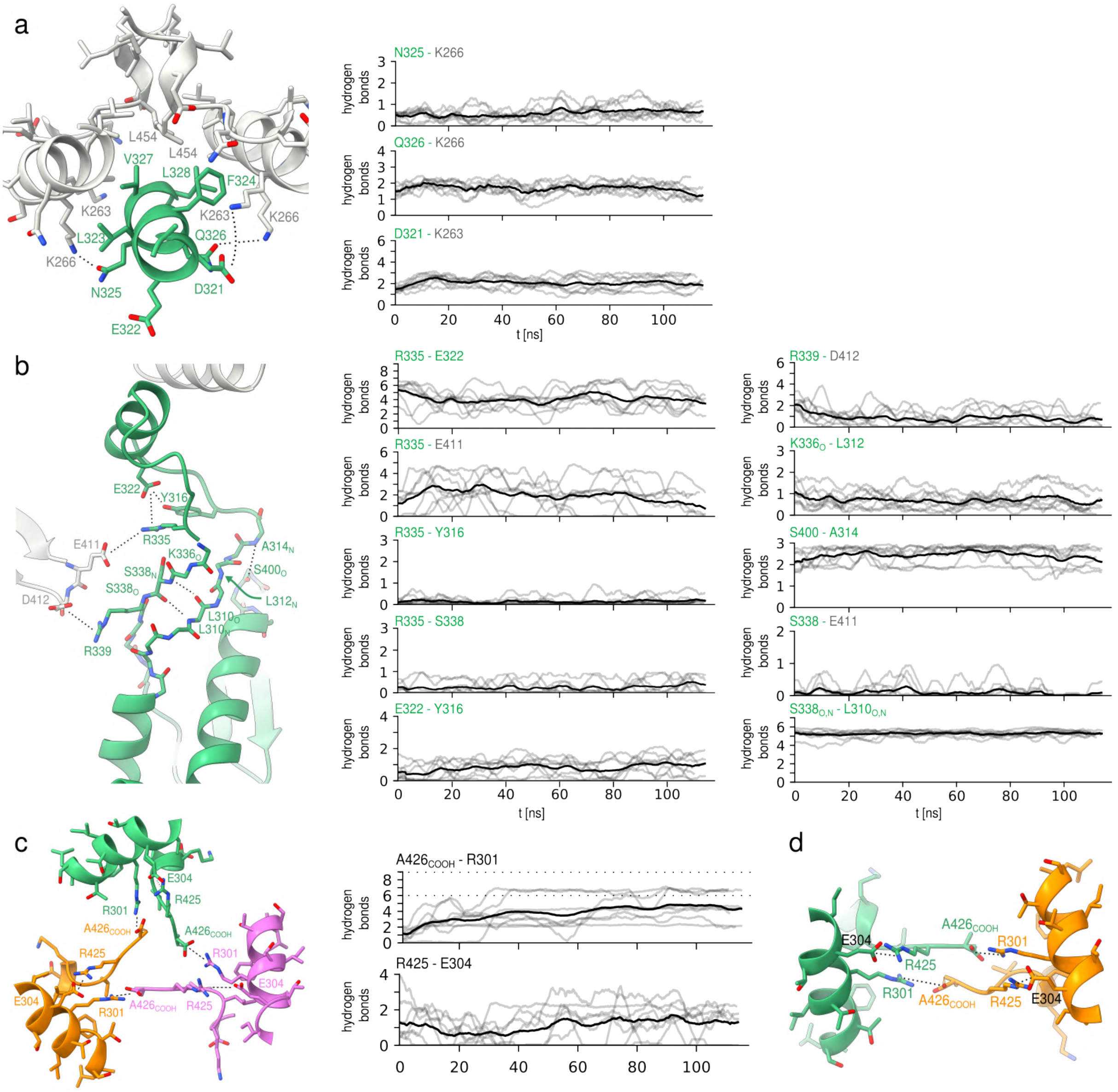

Supplementary figure 5: **Specific interactions observed in molecular dynamics simulations.** **a**, The M2 motif (green) within the E2 CTD binding pocket. Plots denote the number of observed interactions between the indicated residues in each simulation (which has 3 copies of each interaction), with a rolling average of 2ns. In all plots, transparent traces indicate one of 8 replica simulations, and the black solid trace represents the average at that time-point. **b**, The M2-loop region which anchors the CBD trimer to the PDC core is stabilized by a number of backbone interactions, and its conformation is apparently partially stabilized by side-chain mediated polar interactions. **c**, The homomeric E3BP interface of the CBD trimer, seen from above, showing interactions frequently observed in simulations that stabilize the C-terminus and form domain-swapping. Color as in Fig S2B. **d**, The same interactions as in panel C, observed in simulations where one CBD monomer was omitted, thus forming a 2-fold interface with similar properties.

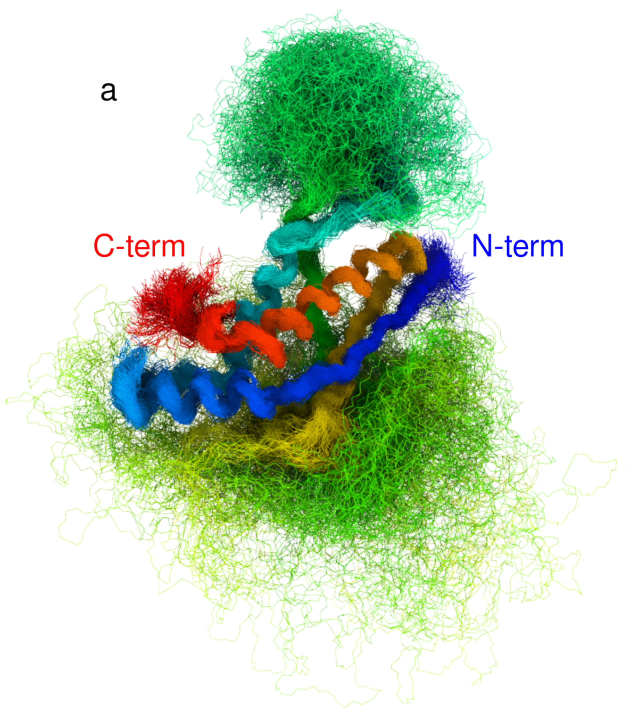

Supplementary figure 6: **Conformation differences of the CBD in simulation.** In both panels, structures are snapshots of the CBD backbone, equally and evenly spaced along simulated time, colored by residue index. **a**, Monomeric CBD simulated in solution. The M3-containing loop of the E3BP CBD remains unstructured on the examined timescales, and show infrequent interactions with the hydrophobic patch of the CBD which forms the homomeric trimer contact. Structures are sampled from 23 replica simulations of monomeric CBD, with each replica simulated for 100 ns (2.3 us total). **b**, Trimeric CBD in complex with the PDC core. The homomeric interface is not accessible, which displaces the M3-loop from a possible such interaction. The M2-containing loop also shows decreased flexibility relative to the core fold, owing to its interaction with the PDC core and the additional restraint imposed by the CBD trimer contact. Structures are sampled from 3 monomers of 8 replica simulations of trimeric CBD, with each replica simulated for at least 100 ns (>2.4 us total).

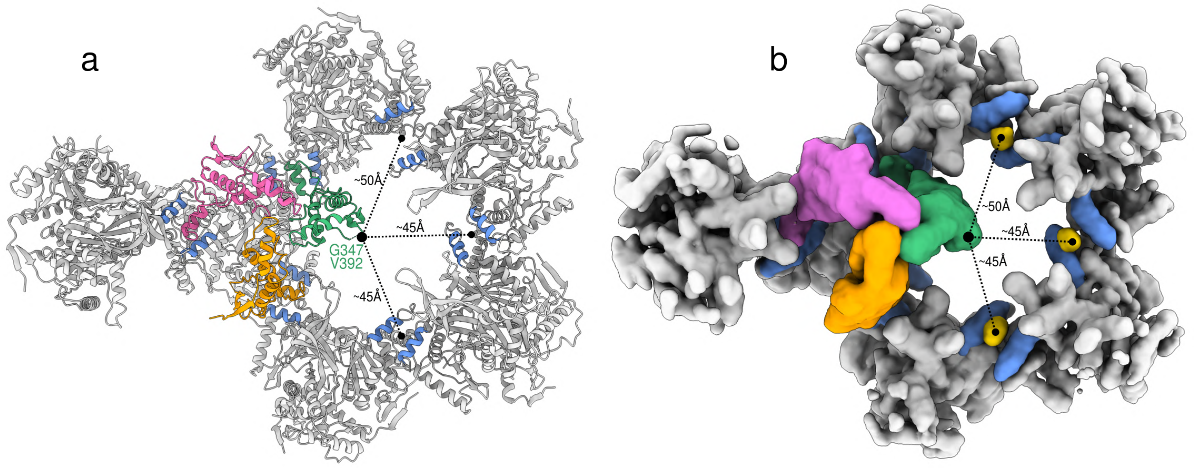

Supplementary figure 7: **Available binding sites in E3BP-occupied PDC cores.** **a**, E3BP residues within the range G347-V392 are not resolved in the present reconstruction due to disorder, in line with prediction. The termini of this stretch however co-localize, approximately to the 5-fold symmetry axis of he PDC core assembly (green). The CBD fold sterically prevents additional E3BP trimers from utilizing any of the remaining 3 binding sites (blue) that are most proximal to both G347 and V392. The approximate distances to each of those three binding sites is shown as dashed lines. **b**, Residual density is observed in these interfaces (yellow), which is attributed to either the M3 motif residing in the disordered region between G347 and V392, or additional non-trimeric E3BP bound through the M2 motif.

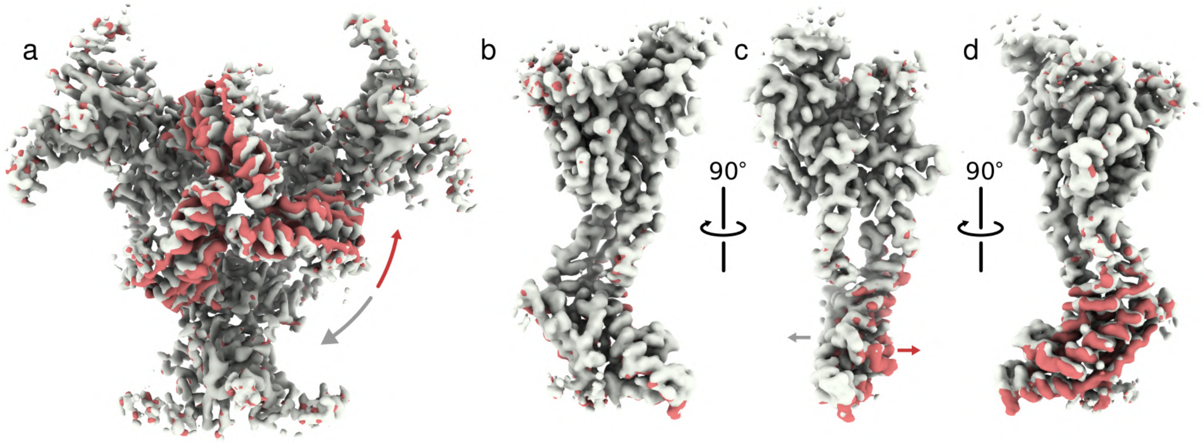

Supplementary figure 8: **Flexibility in E3BP trimers. a**, Particles of E2 trimers with high occupancy of E3BP were classified into 2 classes (gray and red, respectively) without additional alignments and C3 symmetry. The forced symmetry restricts the classifiable range of motion to rotation around the C3-axis and translation along it, but a view from within the core nevertheless shows a range of flexibility of the CBD with respect to the E2 core that will limit attainable resolution **b-d**, Isolating a single E2 monomer and E3BP monomer shows this more clearly. Parametrization of this range of motion is expected to improve the resolution further, but this was not conducted in the present work..

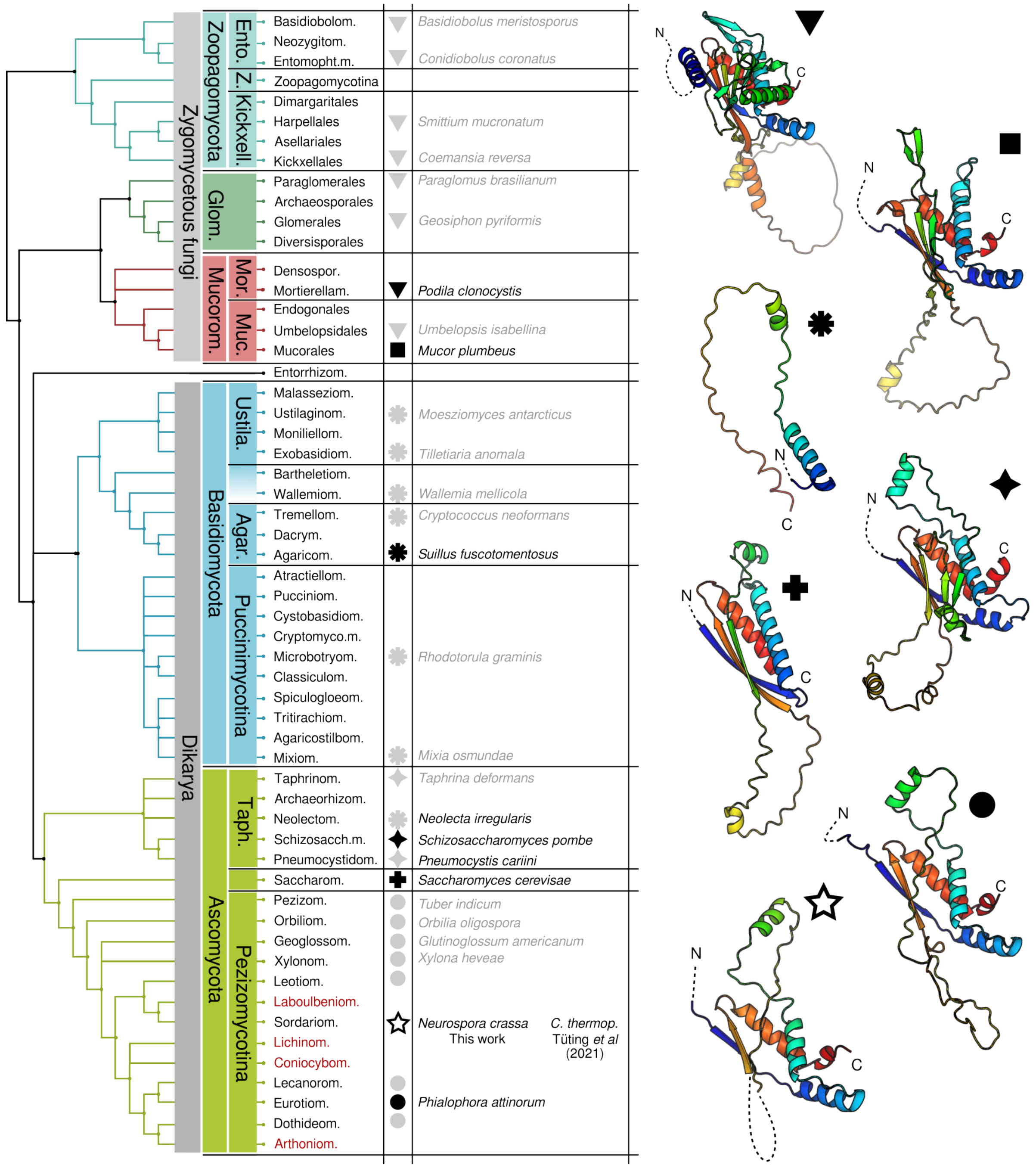

Supplementary figure 9: **Phylogenetic relationship of fungal species and E3BP.** The relationship of fungal species is taken from Naranjo-Ortiz and Gabaldon[4]. Symbols within the table are legends which refer to the most similar E3BP type, as inferred from sequence similarity to models constructed by colabFold. Modeled species are indicated, and black symbols indicate the model shown on the right. A star indicates *N.crassa*, and the structure determined in the present work.

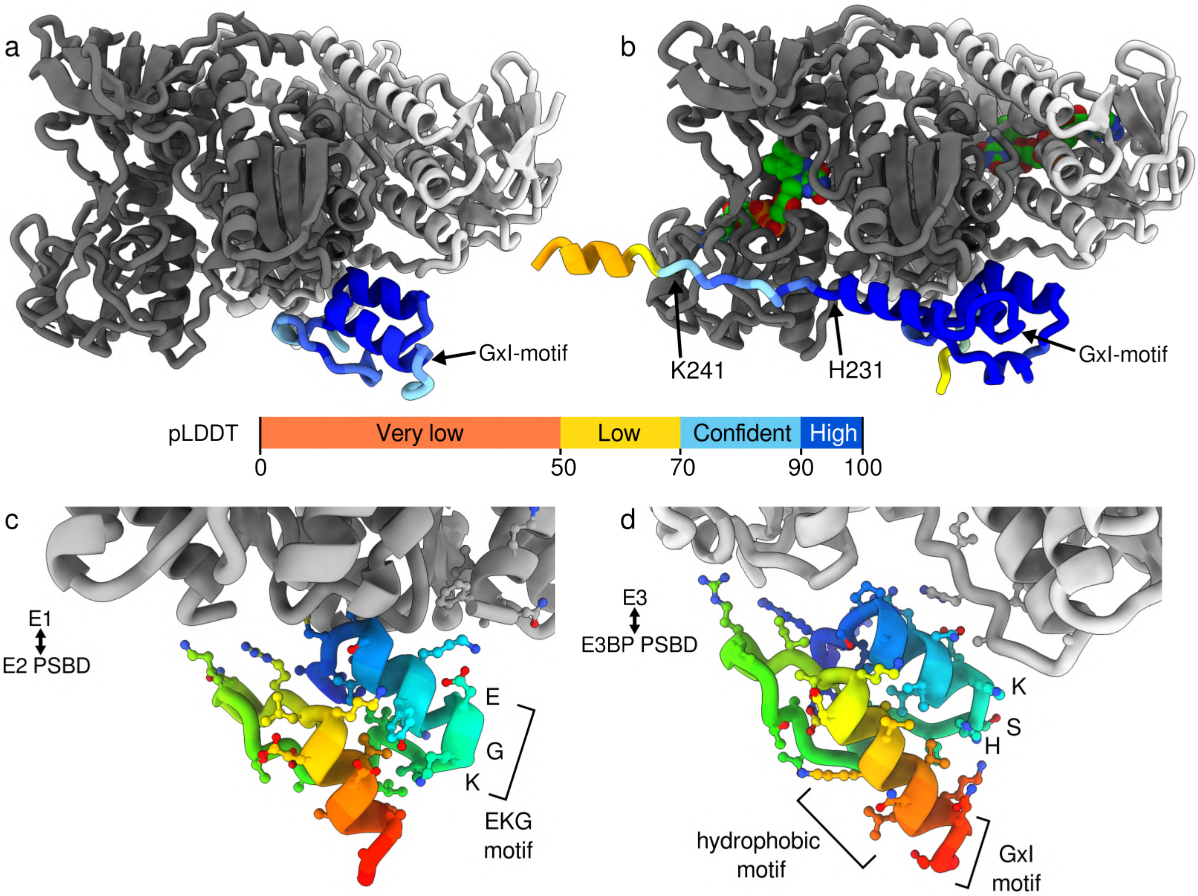

Supplementary figure 10: **Predicted models of PSBD binding.** **a**, Computational prediction of human E3BP:E3 complex. Only the PSBD is colored by pLDDT, since all E3 residues shown have pLDDT>90. E3 monomers are individually colored by white and gray for clarity. The FAD co-factor molecule is shown (green). This model agrees well with present structures and models of binding. **b**, Computational prediction of *N.crassa* E3BP:E3 complex (supp. data AF2.NC.E3-E3BP), colored as in panel A. Downstream of the GxI motif a helical region extends towards the E3 substrate pocket, which is further lined by E3BP residues H231-K241. **c**, Details of the computationally predicted human E1-E2 interface. The EKG-motif is indicated, facing away from the E1 binding interface. This motif is conserved in both animal and fungal E2, but lacking in both animal and fungal E3BP (cf. Fig. 4A-B). **d**, Details of the computationally predicted human E3-E3BP interface. The EKG motifs is not preserved in the PSBD of E3BP, but two additional motifs distinguish it from the PSBD of E2. Both the hydrophobic and GxI motifs also face away from the predicted binding interface, which agrees with previous observations.

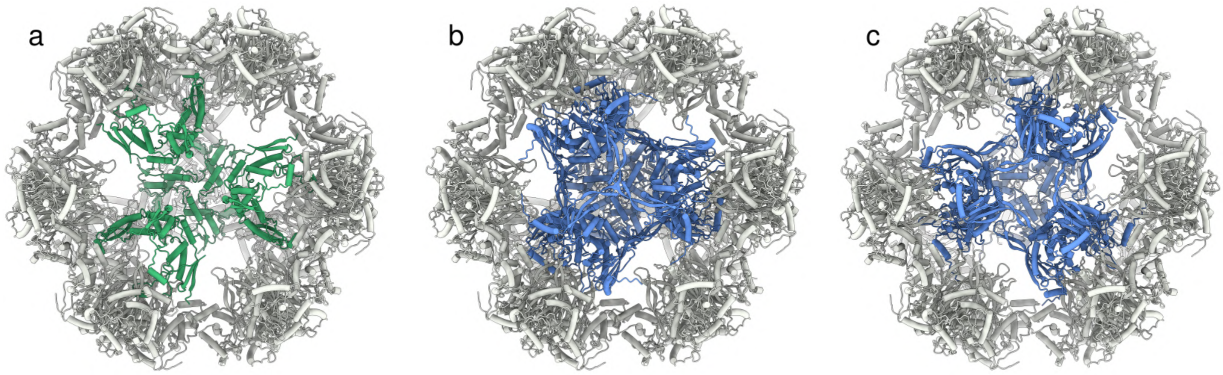

Supplementary figure 11: **Comparison of observed and potential core-internal E3BP domains.** **a**, The E3BP CBD permits 12 internal monomers as 4 trimers. **b**, Superposing the full ancestral E2 fold onto the observed CBD results in minimal steric clashes, limited to the core-internal loop of the superposed E2 copies. The specific arrangement of the trimers interior to the core however matters. In native preparations of *N.crassa* PDC, its CBD was shown to arrange in both of two possible core-internal configurations that maximized occupancy to 12 monomers per core. **c**, The alternate configuration of core-internal CBD results in further reduced clashes, again limited to the very tip of the core-internal loop of the superposed E2 copies. In this configuration, these loops extend not towards each other, but towards the CBD core fold of each other, producing a degree of potential domain-swapping. It is emphasized that panels B and C depict theoretical models under and extended transacetylase fold if it were to occupy a position which aligns with that of the observed E3BP CBD.

Supplementary References

1. Forsberg, B., Aibara, S., Howard, R. J., Mortezaei, N. & Lindahl, E. Arrangement and symmetry of the fungal E3BP-containing core of the pyruvate dehydrogenase complex. *Nat. Commun.* **11,** 1–10 (2020).
2. EMPIAR-10489: Native Pyruvate Dehydrogenase Complex from Neurospora crassa, doi:10.6019/EMPIAR-10489 (July 2020).
3. Zenodo-7801353: Complementary sequence, model and simulation data for fungal E3BP, doi:10.5281/zenodo.7801353 (Mar. 2023).
4. Naranjo-ortiz, M. A. & Gabald, T. Fungal evolution : diversity , taxonomy and phylogeny of the Fungi. *Biol. Rev.* **94,** 2101–2137 (2019).
