# Supplementary material for: The structure and evolutionary diversity of the fungal E3-binding protein
Source: Commun Biol. 2023 May 3;6:480. doi: 10.1038/s42003-023-04854-7 (PMC10156792; doi:10.1038/s42003-023-04854-7)
Supplement: Supplementary file 3 — Description of Additional Supplementary Files [file 42003_2023_4854_MOESM3_ESM.pdf]

Description of Additional Supplementary Files

File name: Supplementary Data 1

Description: Source data for Figure 1e.
